# Methodological Rigor and Transparency in Clinical Practice Guidelines for Nutrition Care in Critically Ill Adults: A Systematic Review Using the AGREE II and AGREE-REX Tools

**DOI:** 10.3390/nu14132603

**Published:** 2022-06-23

**Authors:** John K. Noyahr, Oana A. Tatucu-Babet, Lee-anne S. Chapple, Christopher Jake Barlow, Marianne J. Chapman, Adam M. Deane, Kate Fetterplace, Carol L. Hodgson, Jacinta Winderlich, Andrew A. Udy, Andrea P. Marshall, Emma J. Ridley

**Affiliations:** 1 Australian and New Zealand Intensive Care Research Centre, Department of Epidemiology and Preventative Medicine, School of Public Health and Preventive Medicine, Monash University, Melbourne, VIC 3004, Australia; jknoy1@student.monash.edu (J.K.N.); oana.tatucu@monash.edu (O.A.T.-B.); carol.hodgson@monash.edu (C.L.H.); jacinta.winderlich@monash.edu (J.W.); andrew@udy.com (A.A.U.); 2 Intensive Care Unit, Royal Adelaide Hospital, Adelaide, SA 5000, Australia; lee-anne.chapple@adelaide.edu.au; 3 Adelaide Medical School, The University of Adelaide, Adelaide, SA 5000, Australia; 4 Centre of Research Excellence in Translating Nutritional Science to Good Health, The University of Adelaide, Adelaide, SA 5005, Australia; marianne.chapman@sa.gov.au; 5 Cardiothoracic and Vascular Intensive Care Unit, Auckland City Hospital, Auckland 1023, New Zealand; jake@jakebarlow.me; 6 Department of Medicine and Radiology, Melbourne Medical School, Royal Melbourne Hospital, University of Melbourne, Melbourne, VIC 3010, Australia; adam.deane@mh.org.au (A.M.D.); kate.fetterplace@mh.org.au (K.F.); 7 Department of Allied Health (Clinical Nutrition), Royal Melbourne Hospital, Melbourne, VIC 3050, Australia; 8 Intensive Care Unit, Alfred Hospital, Melbourne, VIC 3004, Australia; 9 Paediatric Intensive Care Unit, Monash Children’s Hospital, Melbourne, VIC 3168, Australia; 10 Intensive Care Unit, Gold Coast Hospital and Health Service, Gold Coast, QLD 4215, Australia; a.marshall@griffith.edu.au; 11 Menzies Health Institute Queensland, Gold Coast Campus, Griffith University, Gold Coast, QLD 4222, Australia; 12 Nutrition Department, The Alfred Hospital, Melbourne, VIC 3004, Australia

**Keywords:** systematic review, practice guideline, critical illness, intensive care units, nutrition therapy, energy metabolism

## Abstract

*Background:* To evaluate the methodological quality of (1) clinical practice guidelines (CPGs) that inform nutrition care in critically ill adults using the AGREE II tool and (2) CPG recommendations for determining energy expenditure using the AGREE-REX tool. *Methods:* CPGs by a professional society or academic group, intended to guide nutrition care in critically ill adults, that used a systematic literature search and rated the evidence were included. Four databases and grey literature were searched from January 2011 to 19 January 2022. Five investigators assessed the methodological quality of CPGs and recommendations specific to energy expenditure determination. Scaled domain scores were calculated for AGREE II and a scaled total score for AGREE-REX. Data are presented as medians (interquartile range). *Results:* Eleven CPGs were included. Highest scoring domains for AGREE II were clarity of presentation (82% [76–87%]) and scope and purpose (78% [66–83%]). Lowest scoring domains were applicability (37% [32–42%]) and stakeholder involvement (46% [33–51%]). Eight (73%) CPGs provided recommendations relating to energy expenditure determination; scores were low overall (37% [36–40%]) and across individual domains. *Conclusions:* Nutrition CPGs for critically ill patients are developed using systematic methods but lack engagement with key stakeholders and guidance to support application. The quality of energy expenditure determination recommendations is low.

## 1. Introduction 

Clinical practice guidelines (CPGs) evaluate the existing literature to determine a set of recommendations that consider both the benefits and harms of different therapeutic options [1]. Despite the wide availability and acceptance of formal processes for development, CPGs vary in quality, and the level of evidence available to inform their recommendations ranges from expert opinion and consensus to robust randomized controlled trials (RCTs) [2]. 

In critical care nutrition, definitive research has not been conducted in several key recommendation areas [3]; therefore, CPGs frequently contain consensus statements that are based on expert opinion of “expected” best practice. Understanding the development process for CPGs in specific fields of practice and the recommendations within them provides clinicians with a clearer understanding of the robustness that practice recommendations are built on, and better informs practice. One core area of practice is the method used to determine energy expenditure in critically ill patients. This is of particular interest as the method selected to estimate (e.g., using predictive equations) or measure (e.g., using indirect calorimetry) energy expenditure guides energy delivery throughout ICU admission, which may increase under- and overfeeding and affect clinical outcomes. Energy expenditure determination is challenging in the ICU and is particularly difficult in patients with obesity, a subpopulation which represent approximately 16% of ICU admissions, due to variations in body composition that are not quantified routinely in clinical practice [4,5,6]. 

The Appraisal of Guidelines Research and Evaluation (AGREE II) and the newly adapted Appraisal of Guidelines Research and Evaluation—Recommendations Excellence (AGREE-REX) are two validated tools that provide complementary assessment of the methodological quality of CPGs and the included recommendations [7,8]. Previous reviews have highlighted that discrepancies exist in recommendations made between critical care nutrition CPGs, which can be challenging and confusing to clinicians [3,9]. Assessment of the quality of CPGs available in critical care nutrition is important to provide clinicians with an understanding of the rigor underpinning the development of CPGs as well as the research used to generate practice recommendations.

The primary aim of this review was to evaluate the methodological quality of CPGs that inform nutrition care in critically ill adults using the AGREE II tool to guide future CPG development. The two secondary objectives were to use the AGREE-REX tool to assess the recommendations for determining energy expenditure (measured and predicted) in (1) general critically ill patients and (2) critically ill patients with obesity. 

## 2. Methods

A systematic review protocol was finalized a priori according to the Preferred Reporting Items for Systematic Reviews and Meta-Analyses (PRISMA) 2020 statement and registered on PROSPERO (PROSPERO identification CRD42021279930) [10]. 

### 2.1. Eligibility Criteria

Inclusion:

The publication was:

1. A CPG intended to guide nutrition care in a critically ill adult population within the intensive care unit (ICU);
2. Developed via a systematic search of the literature and included an accepted system to rate the level of evidence and;
3. Developed by a professional society or academic group.

Exclusion:

1. Previous versions of the same guideline (if recommendations on a similar topic were included);
2. Guidelines that focus on the recovery, rehabilitation, or post-ICU period only;
3. Guidelines or independent CPG recommendations that are specific to patients with COVID-19;
4. Published prior to 2011.

### 2.2. Information Sources and Search Strategy

The search was developed based on previous database searches conducted by members of the review team and an experienced medical librarian with expertise in literature searches for systematic reviews [11,12]. Database searches of Medical Literature Analysis and Retrieval System Online (MEDLINE) Epub ahead of print via Ovid SP, Excerpta Medica Database (Embase) via Ovid SP, Cumulative Index to Nursing and Allied Health Literature (CINAHL) via EBSCOhost, and Cochrane Database of Systematic Reviews via Wiley were undertaken from 1st January 2011 to 19th January 2022 (Supplementary Table S1 provides the final search strategy for MEDLINE from which all other searches were modified). Additionally, guideline repositories including the CPG Infobase, Guidelines International Network, National Institute for Health and Care Excellence (NICE), Scottish Intercollegiate Guidelines Network, and the National Guideline Clearinghouse were searched. Webpages of national dietetic associations that are members of the International Confederation of Dietetic Associations and national intensive care societies listed on the European Society of Intensive Care Medicine (ESICM) webpage were searched for publicly available content. Clinical practice guidelines not available in English were excluded. 

### 2.3. Selection Process

All retrieved articles were uploaded to EndNote, where one assessor (J.K.N) removed duplicates and obviously irrelevant articles using a previously determined systematic cleaning process developed by the authors and modified specifically for this review (Supplementary Figure S1). Following this, two assessors (J.K.N and O.A.T.-B.) used Covidence (Covidence systematic review software, Veritas Health Innovation, Melbourne, Australia; available at www.covidence.org, accessed on 22 February 2022) to independently screen the titles and abstracts of the articles against the eligibility criteria. The full text of included articles was then screened against the same criteria and the discrepancies resolved through discussion with a third assessor (E.J.R.). The authors of the articles were not contacted to request additional material. 

### 2.4. Data Collection Process and Collation of Materials 

All supplementary material relating to the methodology of the CPG development was retrieved by one assessor (J.K.N. or E.J.R.) for included articles. CPG characteristics and specific recommendations for energy expenditure determination in both general and critically ill patients with obesity (if included) were extracted by one assessor (J.K.N.) and cross-checked for accuracy by a second assessor (O.A.T.-B.).

### 2.5. Quality Assessment

Each CPG and relevant recommendations were independently assessed by five assessors (J.K.N., L.S.C., C.J.B., A.M.D., E.J.R.), as recommended in the AGREE REX instructions to ensure reliability [13]. For consistency, the same five assessors also completed the AGREE II tool (which recommends a minimum of two assessors). Prior to commencement, each assessor completed the recommended training modules and read the guidance on how to assess each item in the AGREE II and AGREE-REX user manuals [13,14]. Following this, a CPG was chosen as a pilot and a meeting held to discuss items that varied more than 20% in score across the assessors as well as any other aspects that required clarification. 

The methodological quality of the included CPGs was assessed with the AGREE II tool which has 25 items in total (23 items organized into six domains and the final two items used for global rating). A summary of the AGREE II domains is provided in Supplementary Figure S2. The “My AGREE Plus” online platform was used by all assessors to document responses for the AGREE II tool [14]. 

The methodological quality of CPG recommendations for energy expenditure determination within guidelines was assessed using the AGREE-REX tool which has nine items (divided into three domains). An additional and optional item required the assessor to consider whether they would endorse the CPG recommendation for use in (1) the appropriate context and (2) their context; these items were not completed for this review. A summary of the AGREE REX domains is provided in Supplementary Figure S3. 

Items in both the AGREE II and AGREE-REX tool were assessed as recommended on a Likert scale ranging from one (strongly disagree) to seven (strongly agree). 

### 2.6. Synthesis of Results

As recommended in the user manual for both AGREE II and AGREE-REX, a scaled score was calculated for each domain by summing the assessor scores for each of the items in a domain and scaling the total as a percentage of the maximum possible score for that domain using the following formula:

$$Scaled\;Domain\;Score=\frac{Obtained\;Score-Miniumum\;Possible\;Score}{Maximum\;Possible\;Score-Minimum\;Possible\;Score}\times 100$$

The minimum and maximum possible scores were calculated by multiplying one (strongly disagree) or seven (strongly agree) by the number of items in the domain (i.e., three for Domain 1) and number of assessors (five for this review), respectively [14]. It is not recommended to calculate a total scaled score across all domains for the AGREE II tool [7].

For the AGREE-REX tool, scaled domain scores and an overall scaled score (as recommended), using the same scaling method as presented above for the AGREE II score, was calculated [13].

Descriptive statistics (median (interquartile range)) are used to describe scaled domain percentages, item scores, and overall scaled score across the included CPGs and recommendations. Scaled domain percentages, item scores, and overall scaled scores are also presented in tertiles to provide readers with an indication of performance across included CPGs. 

### 2.7. Intraclass Correlation (ICC) Analysis 

Intraclass correlation (ICC) analyses were conducted to assess the agreement between assessors overall and for each of the CPGs when using the AGREE II tool to address the primary objective. The level of agreement was assessed as slight (0.00 to 0.20), fair (0.21 to 0.40), moderate (0.41 to 0.60), substantial (0.61 to 0.80), and very good (0.81 to 1.00) according to previously published criteria [15].

## 3. Results

### 3.1. Study Selection

**Figure 1:**
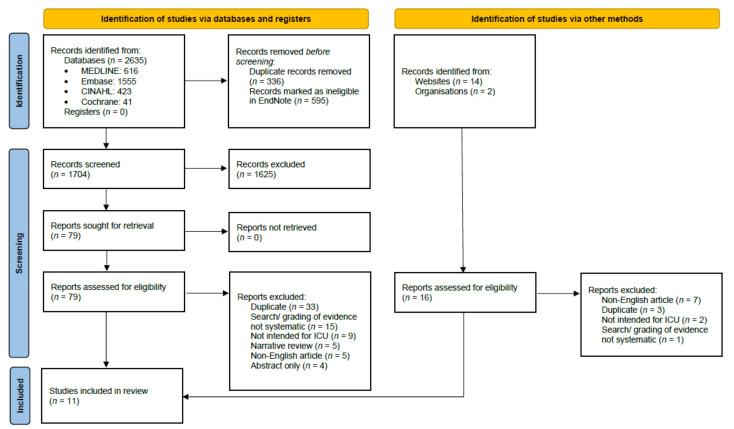
PRISMA flow diagram describing the selection and exclusion of studies.

In total, 2651 articles were identified (*n* = 2635 from database searches and 16 from guideline repositories and professional organization websites). Following the removal of duplicates and irrelevant articles, 1704 articles underwent title and abstract screening, 95 full-text articles were screened, and 11 were included (Figure 1). 

### 3.2. Study Characteristics 

Characteristics of included CPGs are summarized in Table 1. Five (45%) were developed by researchers and clinicians in Europe [16,17,18,19,20], four (36%) in North America [21,22,23,24], and two (18%) in Asia [25,26]. Eight (73%) were published between 2016 and 2021 [16,18,19,21,23,24,25,26] and the remaining three (27%) between 2011 and 2013 [17,20,22]. The GRADE tool was the most common tool used to assess the quality of included evidence and support CPG recommendations (seven (64%)) [16,17,19,20,23,24,26]. 

Eight (73%) CPGs recommended indirect calorimetry as a method for determining energy expenditure in a general ICU population [16,17,18,20,21,22,24,26]. In the absence of indirect calorimetry, CPGs recommended energy expenditure determination via oxygen uptake (VO_2_) or carbon dioxide production (VCO_2_) measurement [16,18], fixed prescriptions (e.g., 25–30 kcal/day) [18,20,24,26], and predictive equations [17,20,22,24,26]. Four (36%) CPGs provided recommendations specific for patients with obesity; all recommended the use of indirect calorimetry where available [18,20,22,24]. Supplementary Table S2 provides the recommendations and level of supporting evidence as stated in each included CPG.

### 3.3. Assessment of CPGs Using the AGREE II Tool 

#### 3.3.1. Domain and Overall Scores

The highest scoring domains for the AGREE II stool were “clarity of presentation” (82% [76–87%]) and “scope and purpose” (78% [66–83%]) and the lowest scoring domains were “applicability” (37% [32–42%]) and “stakeholder involvement” (46% [33–51%]). Domains of “rigor of development” (62% [42–66%]) and “editorial independence” (63% [45–67%]) were considered moderate performing. Table 2 presents the scaled AGREE II domain scores for individual CPGs.

#### 3.3.2. Item Scores

The highest performing item within the AGREE II was item 17, “key recommendations are easily identifiable” (7 [6–7]), while the lowest performing item number was item 5, “the views and preferences of the target population (patients, public, etc.) have been sought” (1 [1–2]). Supplementary Table S3 provides the median item scores by CPG and the raw individual item scores by assessor for AGREE II are presented in Supplementary Figure S4.

#### 3.3.3. Agreement between Assessors with the AGREE II Tool 

Overall, there was “substantial” agreement between assessors across all 11 CPGs (0.66 [0.51–0.72]). Agreement was considered “substantial” for seven CPGs (64%) [17,18,19,21,23,25,26], “moderate” for two (18%) [16,24], and “fair” for the remaining two (18%) [20,22]. 

### 3.4. Assessment of Recommendations Using the AGREE-REX Tool

#### 3.4.1. Domain and Overall Scores 

CPG recommendations for heterogeneous populations were scored as “low” across all individual domains and as a total scaled score (overall score 37% [36–40%]) (Supplementary Table S4). “Clinical applicability” was the highest scoring domain (59% [50–61%]) and the lowest was “values and preferences” (20% [18–23%]). The “implementability” domain was considered moderate performing (40% [30–59%]). Similar results were observed for CPG recommendations relating to energy expenditure determination for patients with obesity (overall scaled score 36% [35–39%]) (Supplementary Table S4). 

#### 3.4.2. Item Scores 

The three highest scoring items (maximum score of seven) were item 1 “evidence” (5 [2–5]), item 2 “applicability to target users” (5 [5–5]) (both within the “clinical applicability” domain), and item 8 “purpose” (within the “implementability” domain) (5 [4–6]) (Supplementary Table S5). The three lowest scoring items fell within the “values and preferences” domain: item 5 “values and preferences of patients/populations” (1 [1–1]), item 6 “values and preferences of policy and decision makers” (1 [1–1]), and item 7 “values and preferences of guideline developers” (1 [1–2]). Similar results were observed for CPG recommendations relating to energy determination for patients with obesity (Supplementary Table S6). The raw individual item scores by the assessor for AGREE-REX are presented in Supplementary Figures S5 and S6.

## 4. Discussion 

This review assessed the methodological quality of CPGs for the nutrition management of critically ill populations using the AGREE II tool and the quality of recommendations specific to energy expenditure determination in critical illness. The highest scoring domains using the AGREE II were “scope and purpose” and “clarity of presentation”, and the lowest were “stakeholder involvement” and “applicability”. Inter-rater reliability varied, but overall indicated substantial agreement between appraisers. The quality of CPG recommendations related to energy expenditure determination was low using the AGREE-REX tool for both general and critically ill patients with obesity. The lowest scoring domain in AGREE-REX was “values and preferences”, especially regarding those of patients, policy/decisions makers, and guideline developers, whilst the highest scoring domain was “clinical applicability”.

The included CPGs scored strongly using the AGREE II tool in the domains of “scope and purpose” followed by “clarity of presentation”. These domains are important because they address aspects that are fundamental to the CPG, including the overall aim, specific health questions, the target population, and language [7]. CPGs written in concise unambiguous language appear to be the most accessed by clinicians and more likely to be implemented [27]. In contrast, domains relating to “Stakeholder involvement” and “Applicability” were infrequently addressed. In particular, the views and preferences of the target population (e.g., patients) were seldomly included in the CPG development process. Although it is now well accepted to involve stakeholders in the CPG development process, there may be several barriers impeding involvement, such as the identification and recruitment of appropriate stakeholders, definition of roles and responsibilities, resources, and time [28,29]. The absence of patients and their unique perspectives may skew the guideline towards specific outcomes that researchers or health professionals value, devising a CPG that may be less meaningful to the patient [30,31,32,33]. Furthermore, keeping patient advocacy groups and consumers up-to-date on developments in practice supports dissemination to target groups and can act as a safeguard against potential conflicts of interest that guideline developers may have [30]. There was also limited engagement of target users, including policy makers. As a result, guideline developers cannot determine whether the CPG answers questions that are relevant to target users from a variety of different settings, including rural and resource-poor settings. It has been shown that not engaging policy makers and other target users may contribute to knowledge waste, where high-quality research is not accessed or used by those for which it was intended and may hinder the dissemination and implementation of CPGs [34,35,36]. The “applicability” domain explores whether CPGs describe facilitators and barriers to uptake, consider resource implications of uptake, and provide tools or advice to support application and monitoring. The identification of barriers is crucial to formulating applicable recommendations [35,36,37,38]. The engagement of target users early on might help to better understand barriers faced when trying to implement guidelines at the individual patient level, healthcare unit level, or organizational level [34,39]. Moreover, very few CPGs considered the economic costs of implementing guidelines, which policy makers use when deciding to implement a guideline into practice, limiting the extent to which system-wide implementation of guidelines might occur [40,41].

Our findings are consistent with systematic reviews by Cattani et al. and Padilla et al. which reported the same highest and lowest scoring domain scores when applying the AGREE II tool to critical care nutrition CPGs [42,43]. Both systematic reviews made recommendations regarding which CPGs were the highest quality and could be used in clinical practice, with the latest Catani et al. review only recommending two guidelines (ESCIM and ADA) for use by clinicians [42,43]. Conversely, the focus of this review was to assess the quality of CPGs using the AGREE II tool to identify areas of improvement and guide future CPG development. 

Recommendations within included CPGs relevant to energy expenditure determination were assessed using the AGREE-REX tool, with “clinical applicability” the highest scoring domain and “values and preferences” the lowest. Many of the development weaknesses that led to low scores on the AGREE-II also contributed to low scores on the AGREE-REX tool. While all guidelines assessed the risk of bias of the included studies using a standardized verified system, such as the GRADE approach, and the direction of the proposed benefit was explicitly stated, most of the evidence used to inform CPG recommendations for energy expenditure determination were of low quality. This can be attributed to the limited number of high-quality RCTs in the area, with most studies comparing indirect calorimetry measurements with predictive equations using an observational study design [3]. It is impossible to remark on the importance of outcomes used and the interpretation of benefits and harms from the consumers’ and other key stakeholders’ perspectives when both were infrequently involved in the development of CPG recommendations; this is a key area for improvement in updates or in the future development of CPG recommendations within the field of critical care nutrition. 

The strengths of this review include using valid and reliable tools, specifically designed to assess the quality of CPGs and their recommendations, and the inclusion of a multidisciplinary team to conduct the appraisals. A systematic methodology was used that can be easily replicated, with a systematic search of four databases as well as online sources of grey literature. Limitations include that the AGREE-II instrument does not suggest cut-off points to define high, moderate, and low quality across domains, so this was interpreted based on tertiles of the domain scores. We only included CPGs that were freely available without a society membership, and it is possible that eligible CPGs may have been missed if they were subscription only. Additionally, although overall agreement across assessors was considered substantial, for some CPGs there was low agreement which may reflect the multidisciplinary team with different clinical experience. However, we purposefully sought a varied team of assessors to reflect those involved in the nutrition care of critically ill patients. 

When applying the included guidelines and recommendations for energy expenditure determination in the clinical setting, it should be considered that, while most guidelines were considered of good quality in terms of methodological rigor and presentation, the lack of patient and key stakeholder input was common. Moreover, the evidence used to inform CPG recommendations for energy expenditure determination is of low quality. Healthcare professionals working in critical care should use the guidelines in conjunction with their own clinical expertise, as well as patient preference, when making decisions about nutrition. Future CPGs would benefit from patient perspectives as well as the engagement of target users, such as a broad range of healthcare professionals and policymakers. One strategy could be to seek feedback from end users in the guideline updating process [35,36].

## 5. Conclusions

Nutrition guidelines for critically ill patients are developed using systematic methods but lack engagement with several key stakeholders, including consumers and tools to support and monitor application. The quality of recommendations related to energy expenditure determination are low for general critically ill patients and those with obesity. 

## Figures and Tables

**Table 1:** Characteristics of included clinical practice guidelines.

Organization/Group Publishing	Title of CPG	Year of Publication	Origin of Contributors	Evidence Grading Method Used	EE General Recommendations	EE Obesity Recommendations
ADA [22]	Critical illness: major recommendations 2012	2012	USA	AAP	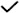	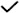
ASPEN [23]	Guidelines for the provision of nutrition support therapy in the adult critically ill patient	2022	USA	GRADE	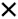	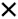
ASPEN/SCCM [24]	Guidelines for the provision and assessment of nutrition support therapy in the adult critically ill patient: SCCM and A.S.P.E.N.	2016	USA	GRADE	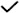	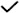
Critical Care Nutrition [21]	Critical care nutrition systematic review	2021	Canada	Own grading system	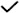	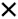
DGEM [18]	Clinical nutrition in critical care medicine—Guideline of the German Society for Nutritional Medicine (DGEM)	2019	Germany	AWMF regulations	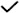	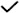
ESCIM [19]	Early enteral nutrition in critically illpatients: ESICM clinical practice guidelines	2017	Europe	GRADE	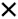	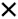
ESPEN [16]	ESPEN guideline on clinical nutrition in the intensive care unit	2019	Europe	GRADE	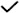	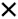
ESPEN Burns [17]	ESPEN endorsed recommendations: nutritional therapy in major burns	2013	France	GRADE	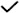	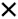
IAB [25]	Practice guidelines for enteral nutrition management in dysglycemic critically ill patients: a relook for Indian scenario	2019	India	Own grading system	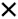	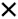
MDA [26]	Medical nutrition therapy (MNT) guidelines for critically ill adults	2017	Malaysia	GRADE	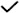	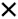
SEMICYUC-SENPE [20]	Guidelines for specialized nutritional and metabolic support in the critically ill patient. Update. Consensus SEMICYUC-SENPE	2011	Spain	GRADE	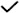	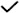

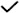
, energy expenditure recommendation included in CPG; 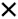, energy expenditure recommendation not included in CPG. Abbreviations: AAP, American Association of Pediatrics; ADA, Academy of Nutrition and Dietetics; ASPEN, American Society for Parenteral and Enteral Nutrition; AWMF, Arbeitsgemeinschaft der Wissenschaftlichen Medizinischen Fachgesellschaften (the Association of the Scientific Medical Societies in Germany); CPG, clinical practice guidelines; DGEM, Deutsche Gesellschaft für Ernährungsmedizin (German Society for Nutritional Medicine); EE, energy expenditure; ESCIM, the European Society of Intensive Care Medicine (ESICM); ESPEN, European Society for Clinical Nutrition and Metabolism; GRADE; Grading of Recommendations, Assessment, Development and Evaluations; IAB, advisory board from nine healthcare centers across India; MDA, Malaysian Dietitians’ Association; SCCM, Society of Critical Care Medicine; SEMICYUC, La Sociedad Española de Medicina Intensiva, Crítica y Unidades Coronarias (Spanish Society of Intensive and Critical Care Medicine and Coronary Units); SENPE, Sociedad Española de Nutrición Clínica y Metabolismo (the Spanish Society of Parenteral and Enteral Nutrition).

**Table 2:** Scaled domain scores using the AGREE II tool (%).

Clinical Practice Guidelines	Scope and Purpose	Stakeholder Involvement	Rigor of Development	Clarity of Presentation	Applicability	Editorial Independence
ADA [22]	78 ^b^	63 ^c^	63 ^b^	87 ^c^	76 ^c^	48 ^a^
ASPEN [23]	69 ^a^	46 ^b^	62 ^b^	60 ^a^	43 ^c^	63 ^b^
ASPEN/SCCM [24]	80 ^b^	64 ^c^	76 ^c^	89 ^c^	40 ^c^	68 ^c^
Critical Care Nutrition [21]	89 ^c^	30 ^a^	66 ^c^	82 ^b^	11 ^a^	5 ^a^
DGEM [18]	82 ^c^	48 ^b^	50 ^b^	81 ^b^	37 ^b^	78 ^c^
ESCIM [19]	84 ^c^	41 ^b^	66 ^c^	84 ^b^	36 ^b^	83 ^c^
ESPEN [16]	76 ^b^	27 ^a^	65 ^b^	76 ^a^	38 ^b^	65 ^b^
ESPEN Burns [17]	46 ^a^	20 ^a^	40 ^a^	87 ^c^	19 ^a^	67 ^c^
IAB [25]	62 ^a^	47 ^b^	26 ^a^	72 ^a^	46 ^c^	17 ^a^
MDA [26]	92 ^c^	53 ^c^	43 ^a^	88 ^c^	37 ^b^	42 ^a^
SEMICYUC-SENPE [20]	54 ^a^	37 ^a^	42 ^a^	76 ^a^	28 ^a^	52 ^b^

Scaled domain scores are presented to indicate the lowest (^a^), middle (^b^), and highest (^c^) tertiles across the six domains of the AGREE II tool. Abbreviations: ADA, Academy of Nutrition and Dietetics; ASPEN, American Society for Parenteral and Enteral Nutrition; DGEM, Deutsche Gesellschaft für Ernährungsmedizin (German Society for Nutritional Medicine); ESCIM, the European Society of Intensive Care Medicine (ESICM); ESPEN, European Society for Clinical Nutrition and Metabolism; IAB, advisory board from nine healthcare centers across India; MDA, Malaysian Dietitians’ Association; SCCM, Society of Critical Care Medicine; SEMICYUC, La Sociedad Española de Medicina Intensiva, Crítica y Unidades Coronarias (Spanish Society of Intensive and Critical Care Medicine and Coronary Units); SENPE, Sociedad Española de Nutrición Clínica y Metabolismo (the Spanish Society of Parenteral and Enteral Nutrition).

## Data Availability

AGREE II and AGREE-REX raw data are included as Supplementary Material.

## Supplementary Materials

The following are available online at https://www.mdpi.com/article/10.3390/nu14132603/s1, Figure S1: Systematic removal of irrelevant articles on EndNote, Figure S2: Summary of the AGREE II domains, Figure S3: Summary of the AGREE-REX domains, Figure S4: AGREE II raw item scores, Figure S5: AGREE-REX raw item scores in general ICU populations, Figure S6: AGREE-REX raw items item scores in critically ill patients with obesity, Table S1: Medline Ovid Search, Table S2: Clinical practice guideline recommendations, Table S3: Median AGREE II item scores for included guidelines from all assessors, Table S4: Scaled domain scores (%) using the AGREE-REX tool for energy expenditure determination in general ICU populations and patients with obesity, Table S5: Median AGREE-REX item scores for energy expenditure in general ICU populations, Table S6: Median AGREE-REX item scores for energy expenditure in critically ill patients with obesity.
