# Supplementary material for: Methodological Rigor and Transparency in Clinical Practice Guidelines for Nutrition Care in Critically Ill Adults: A Systematic Review Using the AGREE II and AGREE-REX Tools
Source: Nutrients. 2022 Jun 23;14(13):2603. doi: 10.3390/nu14132603 (PMC9268338; doi:10.3390/nu14132603)
Supplement: Supplementary file 1 [file nutrients-14-02603-s001.zip › nutrients-1746718-supplementary.pdf]

Supplementary Materials

Figure S1. Systematic removal of irrelevant articles on EndNote.

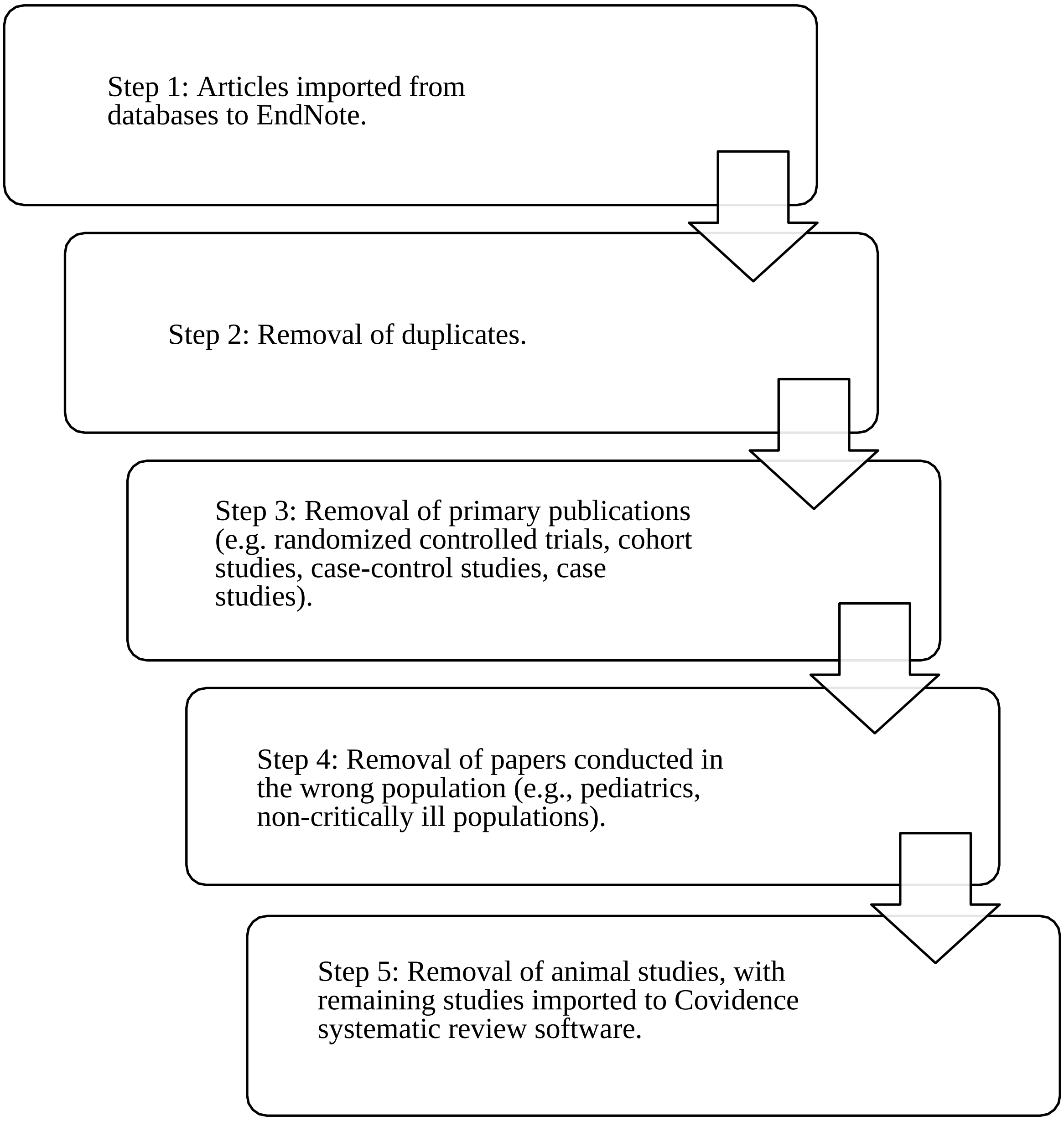

Figure S2. Summary of the AGREE II domains.

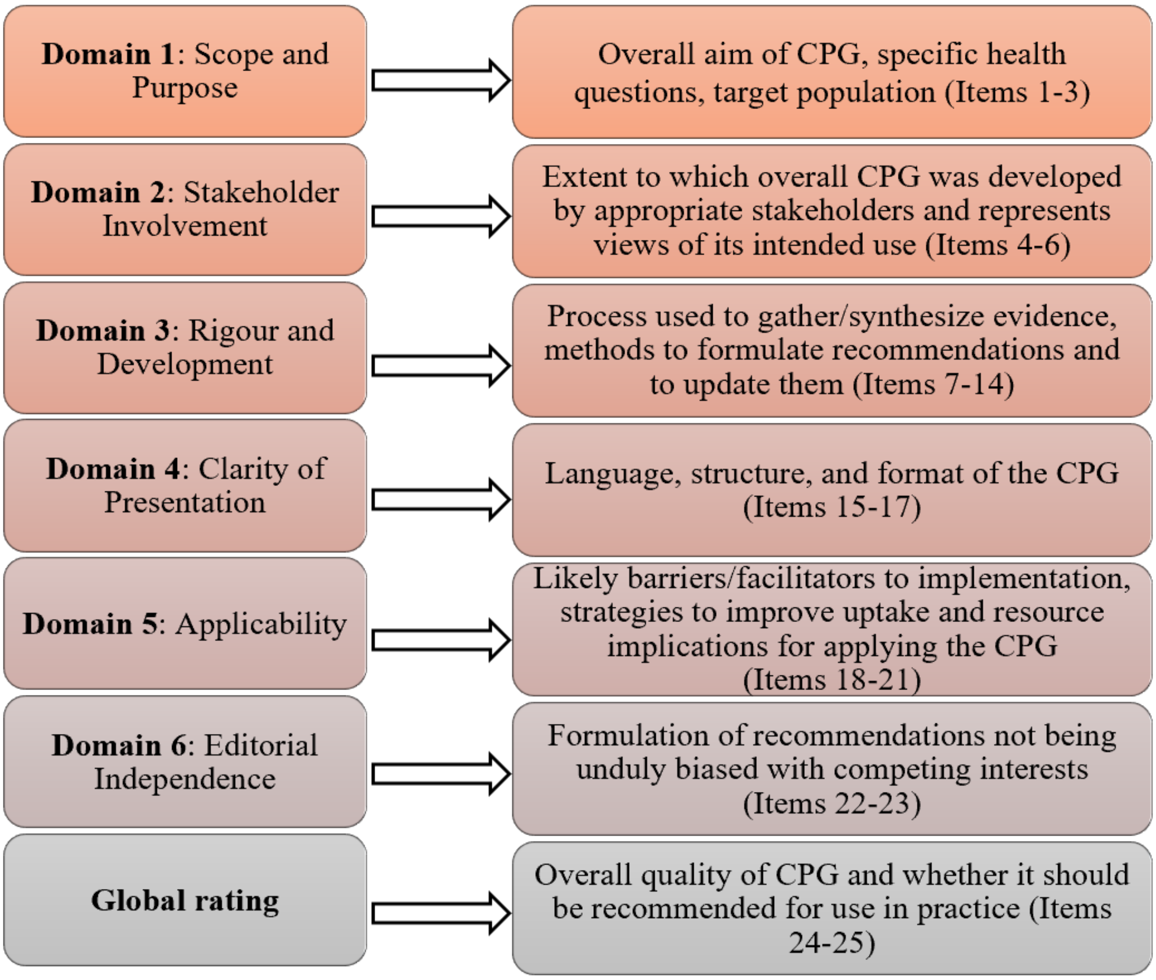

Adapted with permission from Brouwers M, Kho ME, Browman GP, Burgers JS, Cluzeau F, Feder G, Fervers B, Graham ID, Grimshaw J, Hanna S, Littlejohns P, Makarski J, Zitzelsberger L for the AGREE Next Steps Consortium. AGREE II: Advancing guideline development, reporting and evaluation in healthcare. *Can Med Assoc J. 2010.* Available online July 5, 2010. doi:10.1503/cmaj.090449. Abbreviation: CPG, clinical practice guideline.

Figure S3. Summary of the AGREE-REX domains.

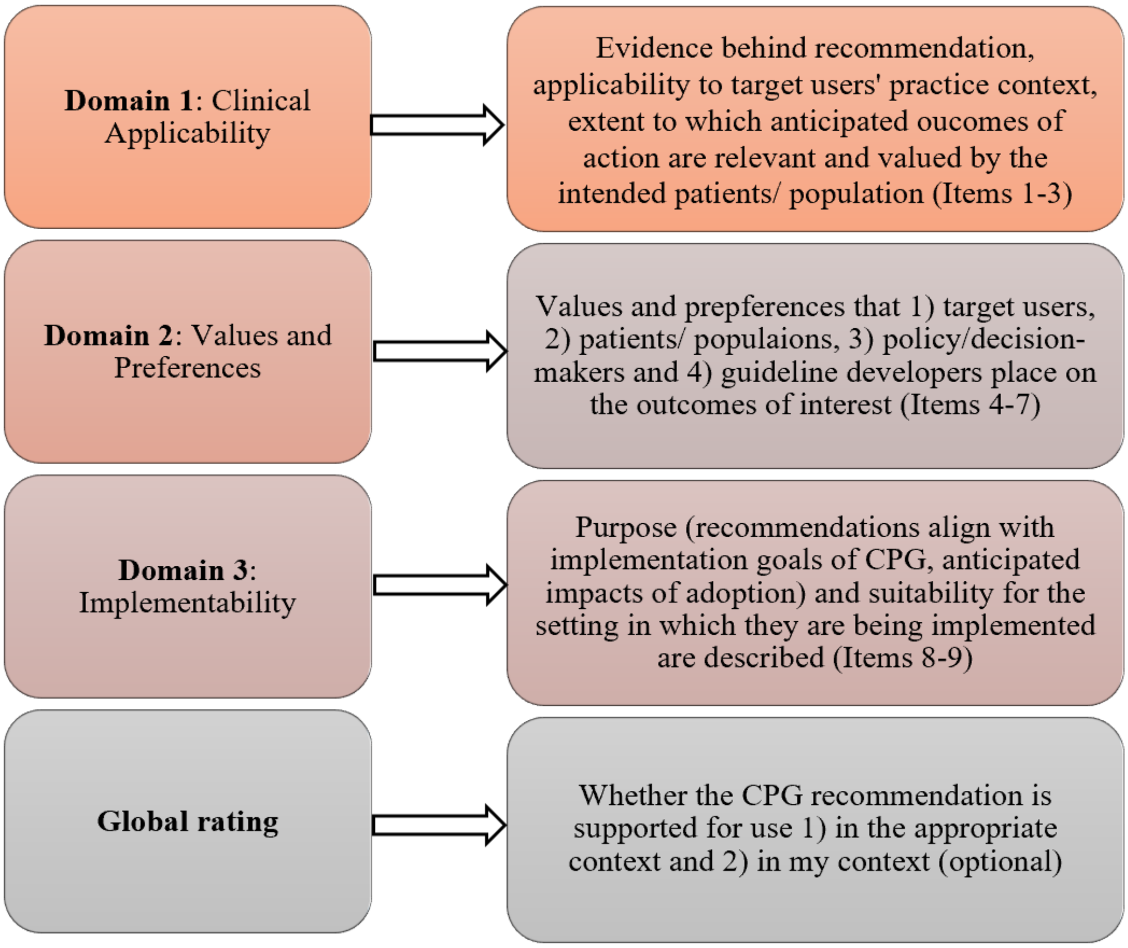

Adapted from Brouwers, M. C., K. Spithoff, K. Kerkvliet, P. Alonso-Coello, J. Burgers, F. Cluzeau, B. Férvers, I. Graham, J. Grimshaw, S. Hanna, M. Kastner, M. Kho, A. Qaseem, S. Straus and I. D. Florez (2020). Development and Validation of a Tool to Assess the Quality of Clinical Practice Guideline Recommendations. JAMA Network Open 3(5): e205535-e205535. Abbreviation: CPG, clinical practice guideline.

Figure S4. AGREE II raw item scores*

Domain	Item	ADA [22]					ASPEN [23]					ASPEN/SCCM [24]					Critical Care Nutrition [21]					DGEM [18]				
		A1	A2	A3	A4	A5	A1	A2	A3	A4	A5	A1	A2	A3	A4	A5	A1	A2	A3	A4	A5	A1	A2	A3	A4	A5
Scope and Purpose	1	7	7	7	3	7	7	5	7	6	6	5	3	4	7	6	6	7	7	7	7	5	7	6	5	7
	2	4	7	5	3	6	7	6	6	7	7	6	4	4	7	7	6	7	3	6	7	6	2	7	6	7
	3	5	6	7	4	7	6	6	7	7	7	7	6	7	7	7	6	6	6	7	7	6	6	5	7	7
Stakeholder Involvement	4	2	2	5	1	5	6	7	6	7	6	7	6	7	6	7	4	6	4	2	6	5	7	4	2	4
	5	6	4	5	3	7	1	1	1	1	1	4	1	1	1	1	4	1	1	1	1	1	1	1	1	1
	6	6	7	5	7	7	6	6	7	7	7	7	4	7	7	7	6	1	1	2	2	6	5	7	6	7
Rigour of Development	7	4	4	2	2	4	7	5	6	7	7	5	4	6	5	5	7	6	7	7	7	3	1	2	1	1
	8	4	4	3	2	4	7	7	6	7	7	5	4	7	4	7	7	6	7	7	7	1	2	2	3	1
	9	4	4	7	3	7	7	7	7	6	7	6	5	7	7	7	6	6	5	6	6	7	4	4	6	6
	10	7	4	5	2	7	4	6	5	7	7	5	4	5	7	7	5	3	4	6	1	6	6	7	7	7
	11	7	4	7	6	7	7	7	6	7	7	5	4	7	7	7	6	4	7	6	1	6	5	6	6	7
	12	5	4	6	5	7	7	7	7	7	7	6	5	7	7	7	7	7	7	6	7	7	6	7	6	3
	13	5	4	5	2	7	3	2	1	1	5	5	3	3	5	4	3	1	1	1	1	2	3	1	2	5
	14	7	6	6	1	7	6	6	6	7	5	6	5	5	6	6	5	1	4	7	1	1	2	5	2	1
Clarity of Presentation	15	7	7	7	6	7	5	5	6	6	6	7	4	7	7	7	6	7	4	5	6	5	5	5	6	7
	16	7	4	7	4	6	4	5	5	5	5	7	6	5	7	7	7	7	7	3	7	7	6	6	5	7
	17	6	6	6	6	7	7	7	7	7	7	6	7	7	4	7	7	6	5	6	6	6	6	4	6	7
Applicability	18	6	6	7	2	7	5	5	2	5	3	1	3	4	5	4	1	2	1	1	1	4	6	1	4	2
	19	7	7	6	4	7	4	3	5	5	5	1	2	4	5	1	1	1	1	1	1	1	2	4	4	1
	20	5	5	5	6	7	5	2	4	5	5	1	6	3	4	1	3	1	4	2	1	2	3	3	4	2
	21	4	6	4	6	4	5	6	4	7	6	5	4	4	6	4	3	2	1	1	4	4	3	4	6	4
Editorial Independence	22	4	6	2	1	6	7	7	3	7	7	4	7	4	6	5	3	1	1	2	1	6	6	4	6	1
	23	6	5	4	1	4	6	5	7	6	6	5	3	6	7	4	1	1	1	1	1	7	7	7	6	7
Overall quality of this guideline	24	6	4	5	2	6	6	6	5	7	7	6	5	7	6	6	6	5	4	5	5	6	5	5	5	4
Recommendation	25	Y	M	M	N	Y	Y	M	M	Y	Y	Y	M	Y	Y	Y	M	Y	Y	Y	M	Y	M	M	Y	Y

Domain	Item	ESICM [19]					ESPEN [16]					ESPEN Burns [17]					IAB [25]					MDA [26]					SEMICYUC-SENPE [20]				
		A1	A2	A3	A4	A5	A1	A2	A3	A4	A5	A1	A2	A3	A4	A5	A1	A2	A3	A4	A5	A1	A2	A3	A4	A5	A1	A2	A3	A4	A5
Scope and Purpose	1	7	4	7	6	7	7	4	5	7	7	3	4	5	4	5	5	5	7	7	6	7	5	7	7	7	5	4	5	2	7
	2	7	5	6	7	7	4	4	7	6	7	4	2	1	3	3	2	4	3	5	5	7	4	5	7	7	6	3	3	4	7
	3	7	4	6	5	6	4	4	4	6	7	5	2	6	5	4	6	2	4	6	4	7	7	7	7	7	4	2	3	2	7
Stakeholder Involvement	4	6	3	3	5	5	3	4	5	2	4	4	3	4	2	4	5	6	6	5	5	3	4	4	4	5	4	3	3	2	4
	5	5	4	1	3	2	1	2	1	1	3	1	1	1	1	1	1	1	1	1	1	1	2	3	2	1	4	1	1	1	1
	6	7	3	2	2	1	3	3	3	3	1	4	1	3	2	1	6	4	4	6	5	7	6	7	7	7	6	1	7	3	7
Rigour of Development	7	7	6	5	7	7	5	4	5	2	7	4	2	2	2	1	4	3	4	2	4	3	2	5	2	3	5	2	5	2	4
	8	7	5	6	6	7	3	3	3	2	5	2	1	1	2	1	1	1	1	1	1	1	1	1	4	1	4	1	1	3	1
	9	6	4	7	6	7	6	6	6	4	7	4	3	4	6	5	4	3	4	2	2	5	2	7	7	3	6	2	7	2	4
	10	6	7	7	5	7	6	5	6	6	7	6	2	7	6	7	4	2	4	4	2	3	1	1	3	1	5	3	7	4	3
	11	6	6	4	6	7	5	5	5	6	7	5	4	5	3	5	4	2	4	5	3	5	2	7	7	3	6	2	7	2	5
	12	7	7	5	7	7	6	6	6	4	7	6	4	6	6	5	4	3	5	3	5	5	3	7	6	5	6	2	7	5	4
	13	1	1	1	1	2	3	2	3	2	4	3	3	5	1	2	1	1	1	1	2	7	3	7	7	7	4	1	4	2	7
	14	1	1	1	1	1	6	6	4	7	5	1	1	1	2	1	1	1	1	1	1	1	1	1	1	1	1	1	1	1	1
Clarity of Presentation	15	7	5	5	7	7	5	5	3	3	7	7	4	4	7	7	5	6	5	5	7	5	4	6	7	7	5	5	4	5	6
	16	6	5	5	5	4	6	5	6	5	7	6	6	7	6	5	6	6	4	2	6	7	6	6	6	5	7	4	7	6	5
	17	7	7	7	7	7	5	7	7	5	7	6	7	7	7	7	4	7	4	6	7	7	7	7	7	7	5	4	6	7	7
Applicability	18	5	4	1	3	1	2	3	4	2	1	1	1	1	2	1	2	2	4	3	1	1	1	3	2	1	4	1	4	2	1
	19	1	1	4	3	1	4	2	5	3	1	1	1	1	3	1	6	6	5	5	3	5	4	7	7	3	1	1	1	4	1
	20	4	5	1	3	5	1	2	5	2	4	1	2	1	1	1	4	3	5	2	1	1	2	4	2	1	2	2	5	3	1
	21	4	4	3	6	4	5	6	4	5	4	5	6	4	5	4	3	6	4	6	4	4	3	4	6	3	4	3	4	5	4
Editorial Independence	22	6	7	5	6	6	4	4	7	5	5	4	7	4	6	3	1	2	3	1	2	1	1	1	4	1	5	7	3	3	4
	23	7	4	5	7	7	2	4	6	7	5	7	6	3	5	5	2	2	4	2	1	7	1	5	7	7	5	3	3	2	6
Overall quality of this guideline	24	7	5	6	7	6	5	5	4	5	6	5	4	4	5	3	4	3	5	2	3	6	3	6	6	3	6	5	4	5	4
Recommendation	25	Y	M	Y	Y	Y	Y	M	M	Y	Y	Y	M	M	M	M	M	M	M	N	N	Y	N	Y	Y	N	Y	Y	M	N	N

* A1-5 = Assessor 1 -5

Item 25: I would recommend this guideline for use. Responses: Y, yes; M, yes with modification; N, no. Abbreviations: ADA, Academy of Nutrition and Dietetics; ASPEN, American Society for Parenteral and Enteral Nutrition; DGEM, Deutsche Gesellschaft für Ernährungsmedizin (German Society for Nutritional Medicine); ESCIM, The European Society of Intensive Care Medicine (ESICM); ESPEN, European Society for Clinical Nutrition and Metabolism; IAB, Advisory board from nine healthcare centres across India; MDA, Malaysian Dietitians'

Association; SCCM, Society of Critical Care Medicine; SEMICYUC, La Sociedad Española de Medicina Intensiva, Crítica y Unidades Coronarias (Spanish Society of Intensive and Critical Care Medicine and Coronary Units); SENPE, Sociedad Española de Nutrición Clínica y Metabolismo (The Spanish Society of Parenteral and Enteral Nutrition).

Figure S5. AGREE-REX raw item scores in general ICU populations.

		ADA [22]					ASPEN/SCCM [24]					Critical Care Nutrition [21]					DGEM [18]					ESPEN [16]					ESPEN Burns [17]					MDA [26]					SEMICYUC-SENPE [20]				
Domain	Item	A1	A2	A3	A4	A5	A1	A2	A3	A4	A5	A1	A2	A3	A4	A5	A1	A2	A3	A4	A5	A1	A2	A3	A4	A5	A1	A2	A3	A4	A5	A1	A2	A3	A4	A5	A1	A2	A3	A4	A5
Clinical applicability	1	5	4	3	5	5	6	5	3	5	6	7	6	6	7	6	6	5	4	5	6	6	5	6	5	6	6	4	3	3	2	5	2	1	6	1	5	3	4	6	4
	2	6	4	7	4	6	6	6	6	4	5	7	4	4	5	6	6	4	5	4	6	6	5	5	5	4	6	5	3	5	2	5	2	4	5	6	5	5	4	5	5
	3	6	1	5	3	4	5	4	2	2	4	6	3	3	3	4	5	4	1	2	7	5	5	1	3	4	5	5	1	2	3	5	2	2	2	4	5	3	2	2	4
Values and preferences	4	5	2	4	6	1	5	5	4	6	2	3	2	2	6	2	5	5	1	2	3	2	3	1	4	2	5	2	2	6	2	3	1	3	6	2	5	4	4	6	2
	5	5	2	4	1	1	1	1	1	2	1	1	1	2	1	1	1	1	2	1	1	2	2	2	1	2	1	1	2	1	1	1	1	5	2	1	1	2	1	2	1
	6	5	1	1	5	1	1	1	1	2	1	1	1	1	2	1	1	1	1	2	1	2	2	1	2	1	2	1	1	2	1	1	1	1	6	1	4	1	1	3	1
	7	4	1	1	6	1	4	1	1	6	1	1	1	1	7	1	3	3	1	6	1	4	3	1	5	1	3	1	1	4	1	1	1	1	7	1	3	1	1	5	1
Implementability	8	6	1	4	6	7	4	1	4	5	7	5	1	6	5	4	5	2	4	6	6	4	1	4	3	4	6	1	4	5	5	6	1	4	7	6	6	1	5	6	6
	9	6	4	6	6	7	2	1	4	6	2	1	1	1	1	1	2	1	3	5	2	3	2	3	2	3	2	1	3	4	1	5	2	6	5	3	5	2	5	6	5

Abbreviations: ADA, Academy of Nutrition and Dietetics; ASPEN, American Society for Parenteral and Enteral Nutrition; DGEM, Deutsche Gesellschaft für Ernährungsmedizin (German Society for Nutritional Medicine); ESPEN, European Society for Clinical Nutrition and Metabolism; MDA, Malaysian Dietitians' Association; SCCM, Society of Critical Care Medicine; SEMICYUC, La Sociedad Española de Medicina Intensiva, Crítica y Unidades Coronarias (Spanish Society of Intensive and Critical Care Medicine and Coronary Units); SENPE, Sociedad Española de Nutrición Clínica y Metabolismo (The Spanish Society of Parenteral and Enteral Nutrition).

Figure S6. AGREE-REX raw items item scores in critically ill patients with obesity.

		ADA [22]*					ASPEN/SCCM [24]					Critical Care Nutrition [21]*					DGEM [18]					ESPEN [16]*					ESPEN Burns [17]*					MDA [26]*					SEMICYUC-SENPE [20]				
Domain	Item	A1	A2	A3	A4	A5	A1	A2	A3	A4	A5	A1	A2	A3	A4	A5	A1	A2	A3	A4	A5	A1	A2	A3	A4	A5	A1	A2	A3	A4	A5	A1	A2	A3	A4	A5	A1	A2	A3	A4	A5
Clinical applicability	1	5	4	3	5	5	6	4	2	6	6						6	3	3	5	6																5	4	5	5	4
	2	6	4	7	4	6	5	5	6	4	5						6	2	3	4	6																5	4	4	4	5
	3	6	1	5	3	4	5	3	2	2	4						5	2	1	2	7																5	1	2	2	4
Values and preferences	4	5	2	4	6	1	5	3	2	6	2						5	2	1	2	3																5	1	1	6	2
	5	5	2	4	1	1	1	1	1	2	1						1	1	2	1	1																1	1	1	2	1
	6	5	1	1	5	1	1	1	1	2	1						1	1	1	2	1																4	1	1	3	1
	7	4	1	1	6	1	4	1	1	6	1						3	1	1	6	1																3	1	1	5	1
Implementability	8	6	1	4	6	7	4	1	4	5	7						5	1	4	6	6																6	1	5	6	6
	9	6	4	6	6	7	2	1	1	6	2						2	1	1	5	2																3	1	1	6	5

* CPG recommendations for the determination of energy expenditure in patients with obesity were not available.

Abbreviations: ADA, Academy of Nutrition and Dietetics; ASPEN, American Society for Parenteral and Enteral Nutrition; DGEM, Deutsche Gesellschaft für Ernährungsmedizin (German Society for Nutritional Medicine); ESPEN, European Society for Clinical Nutrition and Metabolism; MDA, Malaysian Dietitians' Association; SCCM, Society of Critical Care Medicine; SEMICYUC, La Sociedad Española de Medicina Intensiva, Crítica y Unidades Coronarias (Spanish Society of Intensive and Critical Care Medicine and Coronary Units); SENPE, Sociedad Española de Nutrición Clínica y Metabolismo (The Spanish Society of Parenteral and Enteral Nutrition).

Table S1. Medline Ovid Search.

#	Searches
1	Intensive Care Units/ or Burn Units/ or Coronary Care Units/ or Recovery Room/ or Respiratory Care Units/ or Critical Care/ or Early Goal-Directed Therapy/ or Critical Care Nursing/ or Critical Illness/ or Respiration, Artificial/ or Ventilators, Mechanical/
2	Shock/ or Multiple Organ Failure/ or Shock, Cardiogenic/ or Shock, Hemorrhagic/ or Shock, Surgical/ or Shock, Traumatic/ or Systemic Inflammatory Response Syndrome/ or Cytokine Release Syndrome/ or Shock, Septic/ or Respiratory Distress Syndrome, Adult/ or Acute Lung Injury/
3	(((intensive or critical) adj3 (care or unit* or illness*)) or ICU or ITU or SICU or GICU or critical* ill* or (mechanical* adj4 ventilat*) or (intensive therapy adj (unit* or ward* or department*))).mp.
4	((artificial* adj2 (respirat* or ventilat*)) or (critical patient* or ventilatory support) or (ventilat* adj4 patient*)).mp.
5	(((multiple organ* or multi-organ or multiorgan) adj (dysfunction or failure*)) or (systemic inflammatory response or septic shock or sepsis syndrome* or respiratory distress syndrome*)).mp.
6	(neurocritical or neuroICU* or neurointensive or APACHE or sequential organ failure or SOFA score* or ((burn? or coronary or cardiac or respiratory or stroke or transplant* or high dependency) adj (care or unit* or ward* or patient*))).mp.
7	(((septic* or sepsis) adj3 patient*) or (acute adj3 (heart or cardiac or respiratory or pulmonary or lung) adj3 (failure or insufficiency))).mp.
8	1 or 2 or 3 or 4 or 5 or 6 or 7
9	Nutritional support/ or nutrition therapy/ or parenteral nutrition/ or parenteral nutrition, total/ or parenteral nutrition solutions/ or enteral nutrition/ or nutrition assessment/
10	((tube* or artificial) adj3 (feed* or food* or fed or formula or nutrition)).mp.
11	(nutrition* support* or nutrition* therap* or nutrition*).mp.
12	9 or 10 or 11
13	guideline/ or practice guideline/ or evidence-based medicine/
14	(practice guideline* or consensus statement* or consensus guideline*).mp.
15	((practic* or evidence* or clinical* or consensus) adj1 (statement* or protocol* or guideline*)).mp.
16	13 or 14 or 15
17	8 and 12 and 16
18	limit 17 to dt=20110101-20211001

Table S2. Clinical practice guideline recommendations*.

a) general ICU populations

Source CPG	Energy expenditure determination recommendation (s)
ADA [22]	• Indirect calorimetry recommended<br>• If indirect calorimetry is not available, the Penn State University [PSU(2003b)] equation in non-obese patients should be used **(Fair, conditional)**
ASPEN/SCCM [24]	• Indirect calorimetry recommended in the absence of variables that affect the accuracy of measurement. **(Quality of Evidence, very Low)**<br>• If indirect calorimetry is not available, a published predictive or a simplistic weight-based equation (25–30 kcal/kg/equation day) be used to determine energy requirements **(Expert consensus)**
Critical Care Nutrition [21]	The use of indirect calorimetry versus predictive equations may reduce overall mortality; has no effect on infections or ventilator associated pneumonia; has no effect on hospital, ICU length of stay, or duration of ventilation; may be associated with improved nutritional intake.
DGEM [18]	• Indirect calorimetry recommended **(Strong consensus, 100%)**<br>• If indirect calorimetry is not available, 24 kcal/kg actual body weight/day should be used **(Consensus, 86%)**. Alternatively, CO_2 production rate (VCO_2 method) may be used **(Consensus, 87.5%)**

ESPEN Burns [17]	• Indirect calorimetry recommended<br>• If indirect calorimetry is not available, the Toronto equation for burn adults **(Grade D, weak)**
ESPEN [16]	• Indirect calorimetry recommended **(Grade B, strong consensus [95% agreement])**<br>• If indirect calorimetry is not available, VO_2 from pulmonary arterial catheter or VCO_2 derived from the ventilator should be used **(Consensus, 82% agreement)**
MDA [26]	• Indirect calorimetry recommended (**Grade B)**<br>• If indirect calorimetry is not available, there is insufficient evidence to recommend any specific predictive equation. The use of any predictive equation or simplistic weight-based equation (25−30 kcal/kg/d) should be based on clinician familiarity, ease of use, and data availability (**Grade C)**
SEMICYUC-SENPE [20]	• Indirect calorimetry recommended **(A)**<br>• The Fick method and estimation methods do not show a good correlation with indirect calorimetry **(B)**<br>• If indirect calorimetry is not available, 25 kcal/kg of current weight/day in patients with a BMI < 30 is recommended **(C)** and the Penn State equation in mechanically ventilated patients is recommended **(B)**

b) patients with obesity

**Source CPG**	**Energy expenditure method recommendation (s)**
ADA [22]	• Indirect calorimetry recommended

	• If indirect calorimetry is not available, the Penn State University PSU(2003b) equation and PSU(2010) equation should be used in patients aged <60 and ≥ 60 years, respectively **(Fair, Conditional)**
ASPEN/SCCM [24]	• Indirect calorimetry recommended **(Expert consensus)**<br>• If indirect calorimetry is not available, weight-based equation 11–14 kcal/kg actual body weight/day for BMI 30–50 and 22–25 kcal/kg ideal body weight/day for BMI > 50 **(Expert consensus)**
DGEM [18]	• Indirect calorimetry recommended **Strong consensus (100%)**<br>• If indirect calorimetry is not available, 11-14 kcal/kg actual body weight/ day for BMI ≥ 30 kg/m^2 and 22-25 kcal/kg ideal body weight/day (BMI > 50 kg/m^2) should be used **(Strong consensus, 100%)**
SEMICYUC- SENPE [20]	• Continuous indirect calorimetry is recommended **(A)**<br>• If indirect is not available, energy expenditure is highly variable which complicates estimation using predictive equations **(C).** The Ireton-Jones 1992 and Penn-State 1998 formulas have the best correlation with indirect calorimetry **(B)**

* Grading of recommendations as per primary publication (see bold text) **Abbreviations**: ADA, Academy of Nutrition and Dietetics; BMI, body mass index; CPG, clinical practice guideline; DGEM, Deutsche Gesellschaft für Ernährungsmedizin (German Society for Nutritional Medicine); ESPEN, European Society for Clinical Nutrition and Metabolism; ICU, Intensive Care Unit; MDA, Malaysian Dietitians' Association; SCCM, Society of Critical Care Medicine; SEMICYUC, La Sociedad Española de Medicina Intensiva, Crítica y Unidades Coronarias (Spanish Society of Intensive and Critical Care Medicine and Coronary Units); SENPE, Sociedad Española de Nutrición Clínica y Metabolismo (The Spanish Society of Parenteral and Enteral Nutrition), VCO_2, carbon dioxide production; VO_2, oxygen uptake.

Table S3. Median AGREE II item scores for included guidelines from all assessors

**Domain**	**Item**	**ADA [22]**	**ASPEN [23]**	**ASPEN/ SCCM [24]**	**Critical Care Nutrition [21]**	**DGEM [18]**	**ESCIM [19]**	**ESPEN [16]**	**ESPEN Burns [17]**	**IAB [25]**	**MDA [26]**	**SEMICYUC- SENPE [20]**
Clinical Applicability	1.	7	6	5	7	6	7	7	4	6	7	5
	2.	5	7	6	6	6	7	6	3	4	7	4
	3.	6	7	7	6	6	6	4	5	4	7	3
Stakeholder Involvement	4.	2	6	7	4	4	5	4	4	5	4	3
	5.	5	1	1	1	1	3	1	1	1	2	1
	6.	7	7	7	2	6	2	3	2	5	7	6
Rigor and Development	7.	4	7	5	7	1	7	5	2	4	3	4
	8.	4	7	5	7	2	6	3	1	1	1	1
	9.	4	7	7	6	6	6	6	4	3	5	4
	10.	5	6	5	4	7	7	6	6	4	1	4
	11.	7	7	7	6	6	6	5	5	4	5	5
	12.	5	7	7	7	6	7	6	6	4	5	5
	13.	5	2	4	1	2	1	3	3	1	7	4
	14.	6	6	6	4	2	1	6	1	1	1	1
Clarity of presentation	15.	7	6	7	6	5	7	5	7	5	6	5
	16.	6	5	7	7	6	5	6	6	6	6	6

	17.	6	7	7	6	6	7	7	7	6	7	6
Applicability	18.	6	5	4	1	4	3	2	1	2	1	2
	19.	7	5	2	1	2	1	3	1	5	5	1
	20.	5	5	3	2	3	4	2	1	3	2	2
	21.	4	6	4	2	4	4	5	5	4	4	4
Editorial independence	22.	4	7	5	1	6	6	5	4	2	1	4
	23.	4	6	5	1	7	7	5	5	2	7	3

Abbreviations: ADA, Academy of Nutrition and Dietetics; ASPEN, American Society for Parenteral and Enteral Nutrition; DGEM, Deutsche Gesellschaft für Ernährungsmedizin (German Society for Nutritional Medicine); ESCIM, The European Society of Intensive Care Medicine (ESICM); ESPEN, European Society for Clinical Nutrition and Metabolism; IAB, Advisory board from nine healthcare centres across India; MDA, Malaysian Dietitians' Association; SCCM, Society of Critical Care Medicine; SEMICYUC, La Sociedad Española de Medicina Intensiva, Crítica y Unidades Coronarias (Spanish Society of Intensive and Critical Care Medicine and Coronary Units); SENPE, Sociedad Española de Nutrición Clínica y Metabolismo (The Spanish Society of Parenteral and Enteral Nutrition).

Table S4. Scaled domain scores (%) using the AGREE-REX tool for energy expenditure determination in general ICU populations and patients with obesity*

Guideline	General ICU population				Patients with obesity			
	Clinical Applicability	Values and Preferences	Implementability	Overall Score	Clinical Applicability	Values and Preferences	Implementability	Overall Score
ADA [22]	59	31	72	49	59	31	72	49
ASPEN/ SCCM [24]	60	23	43	40	56	19	38	36
Critical Care Nut [21][†]	69	15	27	36				
DGEM [18]	61	18	27	38	51	14	38	32
ESPEN [16][†]	62	19	32	36				
ESPEN Burns [17][†]	44	17	37	30				
MDA [26][†]	41	22	58	36				
SEMICYUC- SENPE [20]	52	24	62	42	49	18	50	36

* Scaled domain scores are presented to indicate the lowest (horizontal lines), moderate (grey colour) and highest (trellis pattern) performing CPG recommendations across the three domains of the AGREE-REX tool. † CPG recommendations for the determination of energy expenditure in patients with obesity were not available. Abbreviations: ADA, Academy of Nutrition and Dietetics; ASPEN, American Society for Parenteral and Enteral Nutrition; DGEM, Deutsche Gesellschaft für Ernährungsmedizin (German Society for Nutritional Medicine); ESCIM, The European Society of Intensive Care Medicine (ESICM); ESPEN, European Society for Clinical Nutrition and Metabolism; IAB, Advisory board from nine healthcare centres across India; MDA, Malaysian Dietitians' Association; SCCM, Society of Critical Care Medicine; SEMICYUC, La Sociedad Española de Medicina Intensiva, Crítica y Unidades Coronarias (Spanish Society of Intensive and Critical Care Medicine and Coronary Units); SENPE, Sociedad Española de Nutrición Clínica y Metabolismo (The Spanish Society of Parenteral and Enteral Nutrition).

Table S5. Median AGREE-REX item scores for energy expenditure in general ICU populations.

Domain	Item	ADA [22]	ASPEN/ SCCM [24]	Critical Care Nutrition [21]	DGEM [18]	ESPEN [16]	ESPEN Burns [17]	MDA [26]	SEMICYUC- SENPE [20]
Clinical Applicability	1. Evidence	5	5	6	5	6	3	2	4
	2. Applicability target users	6	6	5	5	5	5	5	5
	3. Applicability to patients/ population	4	4	3	4	4	3	2	3
Values and Preferences	4. Values and preferences of target users	4	5	2	3	2	2	3	4
	5. Values and preferences of patients/population	2	1	1	1	2	1	1	1
	6. Values and preferences of policy/decision makers	1	1	1	1	2	1	1	1
	7. Values and preferences of guideline developers	1	1	1	3	3	1	1	1
Implementability	8. Purpose	6	4	5	5	4	5	6	6
	9. Local application and adoption	6	2	1	2	3	2	5	5

Abbreviations: ADA, Academy of Nutrition and Dietetics; ASPEN, American Society for Parenteral and Enteral Nutrition; DGEM, Deutsche Gesellschaft für Ernährungsmedizin (German Society for Nutritional Medicine); ESPEN, European Society for Clinical Nutrition and Metabolism; MDA, Malaysian Dietitians' Association; SCCM, Society of Critical Care Medicine; SEMICYUC, La Sociedad Española de Medicina Intensiva, Crítica y Unidades Coronarias (Spanish Society of Intensive and Critical Care Medicine and Coronary Units); SENPE, Sociedad Española de Nutrición Clínica y Metabolismo (The Spanish Society of Parenteral and Enteral Nutrition).

Table S6. Median AGREE-REX item scores for energy expenditure in critically ill patients with obesity.

Domain	Item	ADA [22]	ASPEN/ SCCM [24]	DGEM [18]	SEMICYUC- SENPE [20]
Clinical Applicability	1. Evidence	5	6	5	5
	2. Applicability target users	6	5	4	4
	3. Applicability to patients/ population	4	3	2	2
Values and Preferences	4. Values and preferences of target users	4	3	2	2
	5. Values and preferences of patients/population	2	1	1	1
	6. Values and preferences of policy/decision makers	1	1	1	1
	7. Values and preferences of guideline developers	1	1	1	1
Implementability	8. Purpose	6	4	5	6
	9. Local application and adoption	6	2	2	3

Abbreviations: ADA, Academy of Nutrition and Dietetics; ASPEN, American Society for Parenteral and Enteral Nutrition; DGEM, Deutsche Gesellschaft für Ernährungsmedizin (German Society for Nutritional Medicine); ESPEN, European Society for Clinical Nutrition and Metabolism; MDA, Malaysian Dietitians' Association; SCCM, Society of Critical Care Medicine; SEMICYUC, La Sociedad Española de Medicina Intensiva, Crítica y Unidades Coronarias (Spanish Society of Intensive and Critical Care Medicine and Coronary Units); SENPE, Sociedad Española de Nutrición Clínica y Metabolismo (The Spanish Society of Parenteral and Enteral Nutrition).
